# A Rational Risk Policy? Why Path Dependence Matters

**DOI:** 10.3390/e25020202

**Published:** 2023-01-19

**Authors:** Hans Geboers, Benoît Depaire

**Affiliations:** Quantitative Methods, Research Group Business Informatics, Hasselt University, 3500 Hasselt, Belgium

**Keywords:** path dependence, optimal leverage, risk management, Kelly criterion

## Abstract

The Kelly criterion determines optimal bet sizes that maximize long-term growth. While growth is definitely an important consideration, the focus on growth alone can lead to significant drawdowns, leading to psychological discomfort for a risk-taker. Path-dependent risk measures, such as drawdown risk, provide a means to assess the risk of significant portfolio retracements. In this paper, we provide a flexible framework for assessing path dependent risk for a trading or investment operation. Given a certain set of profitable trading characteristics, a risk-taker who maximizes expected growth can still be faced with significant drawdowns to the point where a strategy becomes unsustainable. We demonstrate, through a series of experiments, the importance of path dependent risks in the case of outcomes subject to various return distributions. Based on Monte Carlo simulation, we analyze the medium-term behavior of different cumulative return paths and study the impact of different return outcome distributions. We show that in the case of heavier tailed outcomes, extra care is needed, and optimal might not be so optimal in the end.

## 1. Introduction

In this article, drawdown risk is studied from a portfolio perspective. The first objective is to develop a framework that can generate insights into possible drawdown characteristics for a risk-taker’s portfolio. The second objective is to use this framework and show the relationship between leverage, optimizing for long-term growth and drawdown risk.

In the landscape of risk and return there are two main paradigms within portfolio theory: Markowitz’s parametric mean–variance framework [1] and the Kelly criterion (also labeled the capital growth criterion or the growth optimal portfolio (GOP) Theory) [2,3], which found its origin in information entropy. While the mean–variance theory initially was a one-period static theory, Markowitz recognized the relevance of the geometric mean return from the Kelly criterion [4]. The growth optimal portfolio (GOP) considers investor behavior under multi-period dynamics with typically an infinite investment horizon. (For a discussion of the Kelly criterion see for instance [5,6,7,8,9,10]).) Thorp [11] shows that under certain circumstances, the optimal Kelly portfolio is approximately one of the Markowitz efficient portfolios but that this approximation can break down badly in practice.

When presented with favorable opportunities over time, the most extreme is to risk your entire wealth. However, it seems a bit like Russian roulette if you played the game many times. One can also wager a small proportion of starting capital, which already seems more reasonable than the first suggestion. In between these two propositions lies the proportional solution presented by [2,3], who recommend optimizing the geometric mean of outcomes. Their findings are consistent with those of [12] in 1738 in an influential paper called “Exposition of a New Theory on the Measurement of Risk” in which he advises maximizing the expected logarithmic utility of wealth.

Latane [3] advised using the Kelly approach for investment situations that concern a significant portion of wealth which have a cumulative effect. (In the case of a large number of independent trials, he advised using the simple arithmetic mean.) This approach [2] starts from a probabilistic Markovian framework to determine the growth optimal portfolio. This is equivalent to the choice of strategy that has a greater probability of leading to as much or more wealth than any other different strategy. The choice of this strategy equally leads to the portfolio with the highest geometric mean.

**Figure 1:**
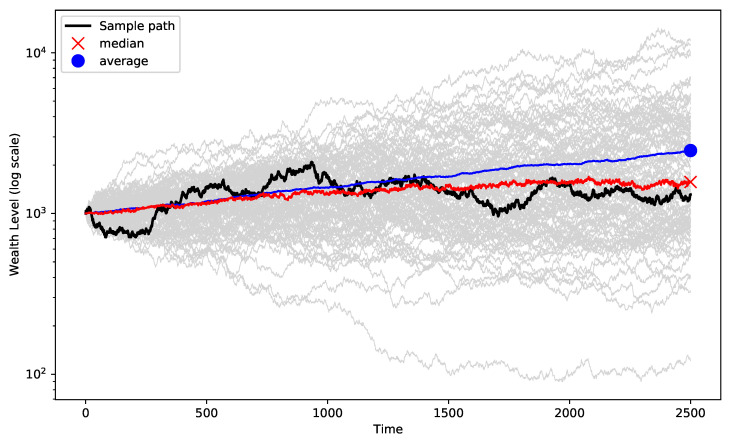
Evolution of wealth over a 10-year period.

As investment and risk always involve a time dimension, the concept of time plays a crucial role in risk-taking. Multiplicative dynamics infer that the future change in the level of wealth is dependent on the current level of wealth. The following example highlights the impact of time on long-term results. Figure 1 shows the evolution of several GBM price paths with a mean of 8% and a volatility of 25% over a period of 10 years. After 10 years we notice a difference between the average level (red dot) and the median level (red cross). This is explained by the fact that a couple of price paths, “the happy few”, are realizing most of the gains. In this case, both the average outcome (2464) and the median outcome (1570) are positive, but the difference is significant. This dynamic can have serious implications for risk-taking and position sizing. (Peters [9] has researched the impact of time on processes that are subject to multiplicative dynamics. For more background on this, see, for example [10].)

The greater the volatility of a certain instrument, the bigger the difference between the geometric and the arithmetic average. This concept has been labeled “volatility drag” by [13]. Assuming portfolio returns follow a normal distribution with arithmetic return μ and volatility σ, the geometric return is given by:

(1) $$\mathrm{Geometric}\;\mathrm{Mean}=\mu -\frac{\sigma^{2}}{2}$$

**Figure 2:**
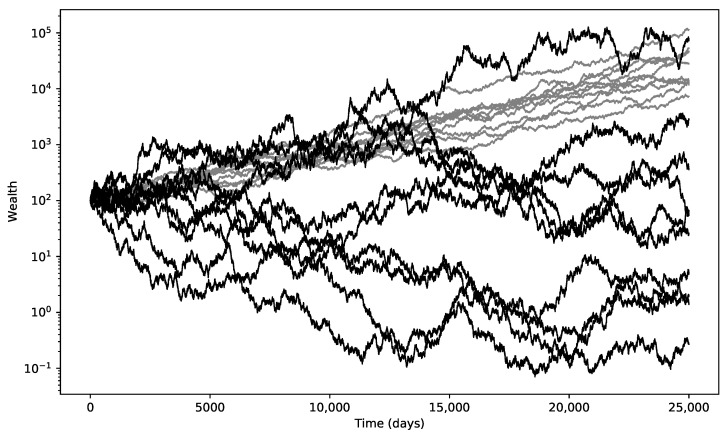
Impact of σ on the long-term growth rate in a stochastic process: 2 different volatility levels (10% and 35%) with a similar expected return of 6% and 10 random price paths for each scenario. The low volatility paths are shown in grey.

The term $\frac{\sigma^{2}}{2}$ is referred to as the volatility drag. It is the hidden tax on an investment portfolio caused by the negative compounding of large investment losses. The higher the volatility, the lower the long-term growth rate, assuming a similar yearly expected return. Figure 2 illustrates this in a stochastic framework. Most of the volatile price paths have a lower terminal value than the lower volatility price paths.

As mentioned in [14,15] it is a reasonable assumption that risk-takers are less concerned with portfolio variations than with the drawdown and path they may face over a time window. While many papers related to bet sizing focus on final outcomes, this paper zooms in on the path that a risk-taker experiences. The objective is to show the importance of randomness in the domain of risk-taking in the case of repeated opportunities subject to a favorable edge. We study path dependent risks starting from the philosophy behind the Kelly growth optimality framework. We therefore develop a framework for measuring portfolio performance under multi-period dynamics. We do not restrict ourselves to the growth optimal bet sizes but also consider fractional Kelly sizing and its implications on portfolio drawdowns.

By means of the drawdown risk measure, we look at the entire account path. Visualizing the impact of different return distributions can give the risk-taker an indication of the type of financial storm to expect. This study shows in general that a higher variance within the return distribution leads to lower levels of optimal risk. This result can also be derived by considering a geometric Brownian motion and calculating the optimal leverage over different levels of variance. The study performed here, however, goes one step further. We show that heavy-tailed return outcomes not only have a big influence on the ultimate growth rate but also have a major influence on the drawdowns experienced by an investor.

This paper is structured as follows: Section 2 provides a review of the literature. In Section 3, we first develop a framework for assessing path-dependent behavior for a trading or investment operation and develop a methodology for the Monte Carlo simulation. We analyze a single performance path and highlight relevant characteristics. In Section 4, we set up a thought experiment. We show how a risk-taker with a positive edge can experience very different outcomes, considering leverage and returns, which are based on a specific probability distribution. Throughout this series of experiments, we show the impact of heavier tails in the return distribution on the drawdown-related performance of a risk-taking operation. We zoom in on the different experiments and study the drawdown behavior based by looking at the maximum loss that is experienced versus a previous high watermark. Section 5 concludes the paper.

## 2. Background and Related Studies

**Figure 3:**
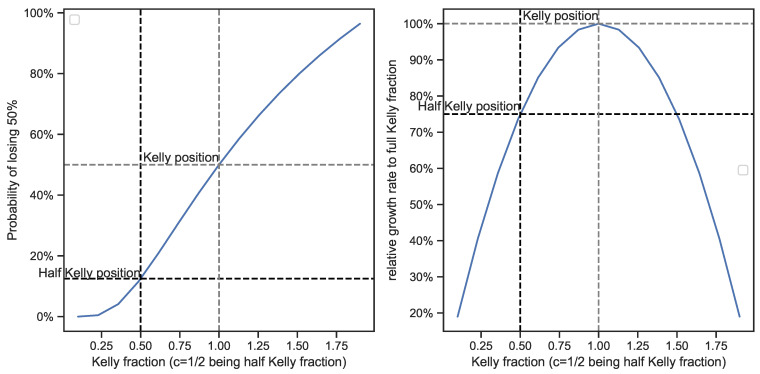
(**Left**): Impact of fractional Kelly on the probability of experiencing a 50% drawdown. (**Right**): Impact of fractional Kelly on relative long-term growth rates.

While the Kelly criterion has many merits as an investment paradigm, one of the major downsides related to this “optimal risk-taking” concept is that it can lead to significant drawdowns [8,16]. An overview of the path-dependent drawdown measures can be found in [17]. The most intuitive approach looks at the maximum loss that a risk taker could have realized versus the high watermark in a specified investment horizon. Within this category, the conditional drawdown (CDD) measure and the conditional expected drawdown (CED) have been distinguished [18]. The CDD measure proposed by [19] includes the maximum drawdown and the average drawdown, which are often used in practice and suitable for portfolio allocation, optimization, and as an input for the $\beta_{CDD}$ measure described in [20,21]. The CED measure developed by [22] allows for a study of the distribution of possible future drawdowns. Thorp [23] worked out several formulas related to drawdowns using the Kelly framework. Assuming a GBM, he came up with an analytical solution for determining the probability of experiencing a drop of X% under full Kelly and fractional Kelly investing. Considering a full Kelly position, the probablity of experiencing a 50% drawdown is 50%. Assuming a half Kelly position, the probability of experiencing a loss of 50% drops to 12.5%, while at the same time, the investor’s long-term growth rate only drops by 25%. These concepts are illustrated in Figure 3. A review of these formulas can be found in Appendix A.1.

Thorp [23] concludes that most people strongly prefer the increased safety and comfort of a fractional Kelly position in exchange for giving up 25% of their growth rate. While there are many assumptions underlying this method, such as the normal distribution of returns and the assumption of the reinvestment of profits, it does show an interesting relationship between risk and growth over time. Several researchers [24,25] also discuss the relationship between Kelly optimal trading and drawdown risks.

Other research on drawdowns related to bet sizing has been performed by [26], who show that for an investor taking Kelly optimal exposure, the average drawdown is just about to diverge. Poundstone [27] refers to the 1/n-rule for an infinite series of serial Kelly bets: the chance of ever dropping to $1/n^{th}$ of your accumulated capital equals 1/n. (This rule is derived from [23] and is based on the assumption that the optimal Kelly fraction = 1). Several papers have addressed the issue of excessive losses related to the Capital Growth Criterion. MacLean et al. [7] provide methods to minimize the probability of ruin and show that, by utilizing fractional Kelly methods, investors can develop a complete trade-off of growth versus security. Lopez de Prado [28] considers growth optimality under a finite time horizon and shows that leverage suggested by growth-optimal portfolio theory needs to be adjusted down considerably. Despite the critiques, the Kelly criterion is still often used as a starting foundation within portfolio selection and optimization. Recent examples can be found in [29,30]. The interesting part about using a theory like the growth optimality criterion, which is linked to information theory, is its intrinsic sensitivity to the non-linear behavior present in financial markets [31].

The asset allocation primer by [32] connects the work of Markowitz, Kelly, and Risk Parity in order to provide a comprehensive overview of different investment strategies and the underlying concepts of portfolio optimization. They explore the specifics of each approach and provide numerical examples to compare asset allocations for the various approaches. Carta and Conversano [33] developed a framework to apply the Kelly criterion to European stock market data and portfolio optimization and showed that the use of the Kelly criterion produced results consistent with the literature.

## 3. Formal Description of the Framework

We model a risk-taking operation based on a Monte Carlo simulation, taking into account the following parameters (These parameters could, for example, be derived from a historical track record of trades.):

- $TR_{i}$ refers to a trade event occurring at time i. The first trade event occurs at the moment i=1.
- *N* refers to the total number of risks taken over the considered period. For example, *N* equals 250 if we model an operation over 5 years with 50 risk events per year.
- $V_{i}$: The cumulative account value at point *i*. $V_{0}$ refers to the initial account value, which equals 1. The cumulative account value $V_{i}$ is considered to be equal to the risk taker’s wealth at point i in the setup of our experiment.
- $IR_{i}$: Initial risk on trade i is expressed as a fraction (in %) of the account value $V_{i-1}$ under the assumption of multiplicative dynamics. For example, if the account value is 1000 and a stock is bought at 100 with a predetermined stop-loss at 90, we consider the IR to be 1% (equivalent to the loss of 10 relative to the account value of 1000). (Under the assumption of additive dynamics, the initial risk on trade *i*, ($IR_{i}$), is expressed as a fraction of the initial account value $V_{0}$.)
- $P_{Win}$ refers to the probability of winning.
- $P_{Loss}$: refers to the probability of losing and is equal to $1-P_{Win}$.
- $RF_{Win}$: The return factor in case of a Win. This return factor is modeled by a specific distribution.
- $RF_{Loss}$: The return factor in case of a Loss. This return factor is modeled by a specific distribution.

**Figure 4:**
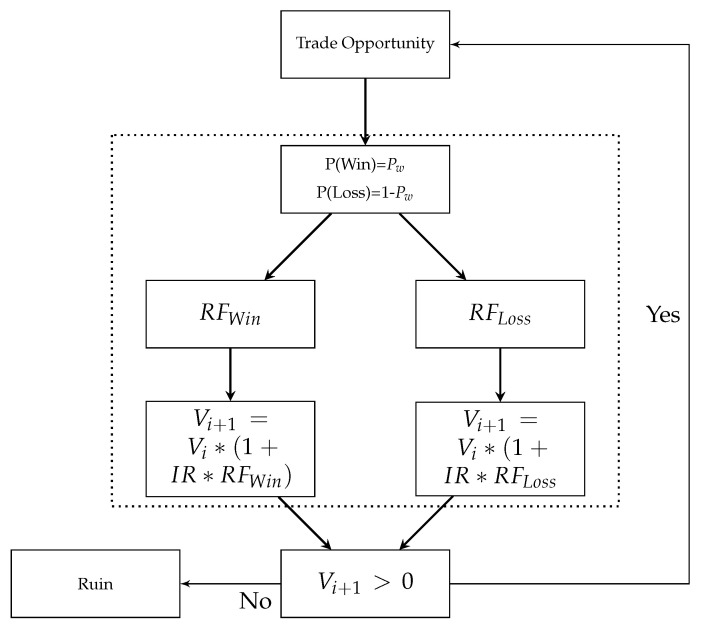
Process of a trade event.

Figure 4 presents the process of a trade event. At first, a trade opportunity presents itself, and an initial risk allocation is assigned to the event. Pre-defining the initial risk is done through a stop-loss at the risk-taker’s discretion and is a function of different variables such as the market under consideration, the volatility, and the liquidity of the product that is being traded. Along with a stop-loss, the risk-taker can assign a target to the position. These are all factors under the control of the risk-taker. The moment the risk is taken, randomness enters the process.

The trade will lead to a profit or a loss. The size of the profit or the loss is determined by the return factor, which is based on a certain probability distribution. One could state that the trade either results in a loss equal to the initial risk, which was determined by the ex-ante stop-loss, or a profit determined by the ex-ante determined target. By assigning a distribution to the return factor, we allow for additional flexibility during a specific trade. For example, a trade might gap through a specific stop-loss, leaving the risk-taker with a much larger loss than expected; a stop-loss may be trailing, leading to a much smaller loss than anticipated by the initial risk; a profit might be taken at a lower level than the target based on certain time or market conditions. Applying a specific distribution function to the return factor provides flexibility in terms of trade outcomes, which in reality might not be the same as the pre-defined risk levels.

After each trade event, the account value *V* is checked. In the event the value is greater than 0, the process of exploiting new trading opportunities is continued. In the event the value is below or equal to 0, the risk taker faces ruin, and risk-taking operations are stopped.

As we are interested in the dynamics of the entire account path, we apply a Monte Carlo simulation to study these dynamics. We consider this path to be a sequence of *N* trading outcomes subject to a random probability distribution. We analyze path-dependent risk by studying the maximum drawdown of the cumulative return path *V*. The maximum drawdown is defined as the maximum peak-to-trough decline of the account value *V* over the course of *N* trading events:

(2) $$\begin{array}{c} MDD\left(V\right)=\underset{i\in [0,N]}{\sup}\underset{s\in [0,i]}{\sup}\left\{\left(V_{s}-V_{i}\right)/V_{s}\right\} \end{array}$$

The conditional expected drawdown (CED) is a risk measure that is derived from these maximum drawdowns. The CED is a practically useful measure of risk as it addresses the expected maximum cumulative asset drop within a given investment horizon. The CED is defined as the tail mean of the maximum drawdown distribution:

(3) $$\begin{array}{c} CED_{\alpha }=E\left(MDD\left(V\right)|MDD\left(V\right)>DT_{\alpha }\right), \end{array}$$

with $DT_{\alpha }$ being the α-quantile of the maximum drawdown distribution. A $CED_{.10}$, for example, refers to the average of the 10% worst drawdowns.

**Figure 5:**
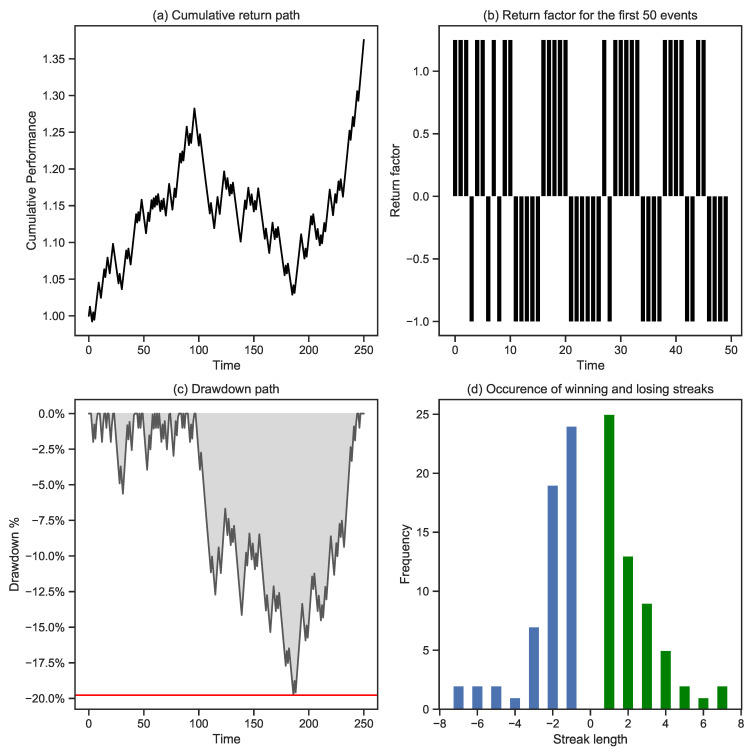
Simulation of one cumulative return path: Panel (**a**) shows the cumulative return path for this specific simulation. Panel (**b**) shows the individual return events: either a win of 1.25% or a loss of −1%. Panel (**c**) shows the drawdown path, with the maximum drawdown, in red, reaching almost 20%. Panel (**d**) is based on an analysis of winning and losing streaks. We note, for example, two occurrences of 7 consecutive losses and two occurrences of 7 consecutive gains. The winning streaks are shown in green, whereas the losing streaks are shown in blue.

Figure 5 provides an example to illustrate the concepts identified above. It shows a simulated cumulative performance path, assuming 250 trading events over a 5-year period, an IR of 1%, a win rate of 50%, and a return outcome of +1.25% in case of a win and −1% in the case of a loss. Panel (a) shows a single simulated initial cumulative return path. Panel (b) shows the returns for each consecutive risk event over time. As defined before, approximately half of the outcomes are wins and half are losses. Another critical metric highlighted in panel (c) is the drawdown path. The drawdown refers to the difference between the highest cumulative performance over a given period and its subsequent value. The maximum drawdown is the largest of all these drawdown values. A thorough analysis of drawdowns provides a comprehensive review of the risks inherent in a trading operation. Finally, we count streaks of consecutive wins and losses in panel (d). There are no surprises in the middle, as there is an almost equal amount of alternating wins and losses. The tails show some interesting observations, such as two sequences of 7 consecutive losses and wins.

## 4. Impact of the Outcome Distribution on Drawdown Risks

Analyzing various outcomes through Monte Carlo simulation can assist in building a rational risk policy that can avoid psychological and financial headaches. Starting from a positive edge, we analyze performance paths based on various risk-taking considerations related to the risk taken per trade, the distribution of trade outcomes, and the probability of winning or losing. We set up several experiments based on a Monte Carlo simulation, keeping the expected return constant. However, the different experiments experience different forms of randomness based on their specific outcome distribution function.

### 4.1. Setup of the Experiment

**Figure 6:**
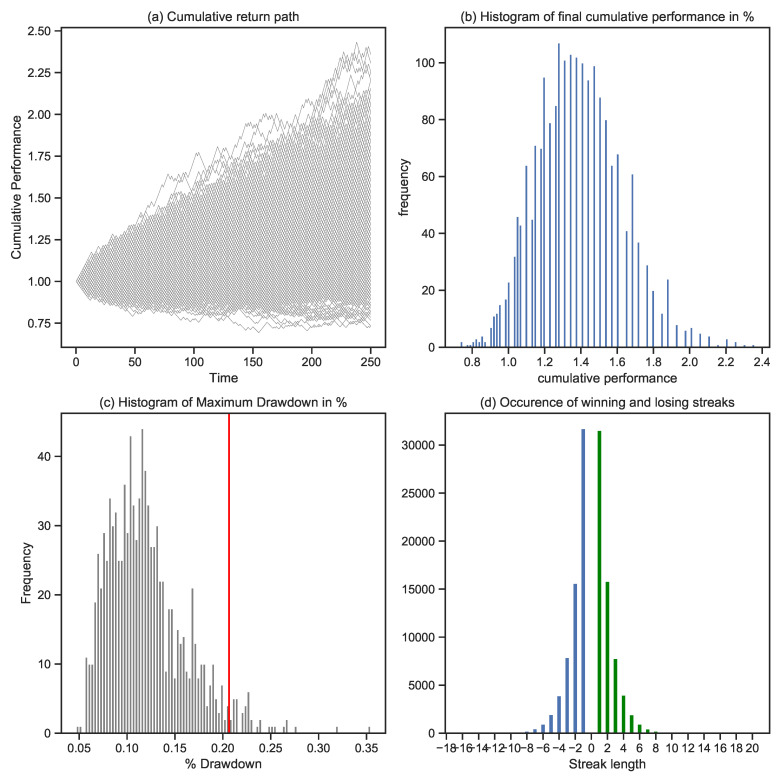
Simulation of 1000 cumulative return paths: Panel (**a**) shows the cumulative return path for each specific simulation. Panel (**b**) shows the distribution of the final outcomes of each simulated process, with most of the final performances ranging between 0 and 80%. Panel (**c**) shows the distribution of the maximum drawdowns related to each performance path; after visual inspection, we note that most of the drawdowns are between 5 and 20%. The red line shows the 10%CED-level. Panel (**d**) is based on an analysis of winning and losing streaks over the entire set of simulations. For each simulation, we count the occurrence and length of losing (blue color) and winning streaks (green color).

Starting from the same parameters used in Figure 5 (IR of 1%, win rate of 50%, and a return outcome of +1.25% in case of a win and −1% in case of a loss), we simulate and analyze 1000 different cumulative performance paths in Figure 6. This figure shows that the exact same risk-taking abilities can lead to various outcomes over a 5-year time frame, purely based on randomness. Each path is made up of N individual risk events. The simulation of several entire paths, based on historical performance statistics, allows us to study different path dynamics that have an impact on the financial and emotional well-being of the risk-taker. Panel (a) shows the cumulative return paths linked to the simulation. Panel (b) shows the distribution of the final performance after 250 events. Panel (c) shows the distribution of the maximum drawdown values of each path and the CED measure, which refers to the tail mean of the maximum drawdown distribution. Panel (d) provides an overview of winning and losing streaks.

In this study, four different scenarios for the distribution function of the return outcomes are considered. Table 1 provides an overview of the distribution of the return outcomes are considered. For each scenario, we study the different return paths by looking at several drawdown and growth statistics for different levels of leverage (measured by IR).

The expected return factor equals 0.125 and will be kept constant throughout this series of experiments. In each scenario, additional uncertainty around the return factor is added by modifying the distribution for the return factor. The probability of winning $P_{w}$ is 50% and equals the probability of losing $P_{l}$ for each experiment.

The first experiment assumes two possible outcomes for the return factor. It is equivalent to the two-point distribution mentioned in the previous section: $RF_{Win}=1.25$ and $RF_{Loss}=-1$. In the second experiment, we assign a uniform distribution to the return factors: $RF_{Win}∼U\left(0,2.5\right)$ and $RF_{Loss}∼U\left(0,-2\right)$. In the third experiment, the uniform distribution is replaced by the exponential distribution.

In the final experiment, we consider a Lomax distribution with shape parameter α and scale parameter λ for the return factor. The shape parameter α of 3 means that the first and second moments are still defined for this distribution. This distribution can be used to model Black Swan events: unexpected and extreme. Lleo and Ziemba [34] provide an example of such an event and discuss the decision of the Swiss National Bank to abandon the peg against the Euro, leading to an increase in the Swiss Franc of more than 20% versus the Euro. These types of events have a significant impact on international investors, banks, and hedge funds.

### 4.2. Results

#### 4.2.1. CASE I: Two-Point Distribution

The results of the simulation for the 2-point distribution for different levels of initial risk can be found in Table 2. Unsurprisingly, we note that the highest median value is achieved at an initial risk of 10%. As the expected value of the return factor is positive, the average level keeps increasing with the amount of leverage or initial risk applied; this happens because there is a very small number of paths that contribute to the majority of the average wealth. For illustration purposes, we consider the expected return at an IR of 25%: 0.5∗0.25∗1.25+0.5∗0.25∗(−1)=3.125%. The expected growth rate over time, also labeled the time-average growth rate, equals: ${\left(1+0.25*1.25\right)}^{0.5}*\left(1+0.25*{\left(-1\right)}^{0.5}\right)-1=-0.78433\%$. (This explains our focus on the median level rather than the average level of the final wealth outcomes for the remaining experiments. Peters [35] provides a thorough discussion on the difference between expected return and time-average growth).

This approach yields results that are mathematically equivalent to the results obtained via the Kelly criterion [2] and the long-term performance criteria suggested by [3]. Applying this criterion to the return factors of 1.25 and −1 yields an optimal initial risk of 10%. As highlighted by [23], this level of initial risk provides a useful upper bound on risk-taking. While this level determines an upper bound for sensible risk-taking, we also show the results if a risk-taker risks more than the upper bound suggested by the growth optimal criterion.

**Figure 7:**
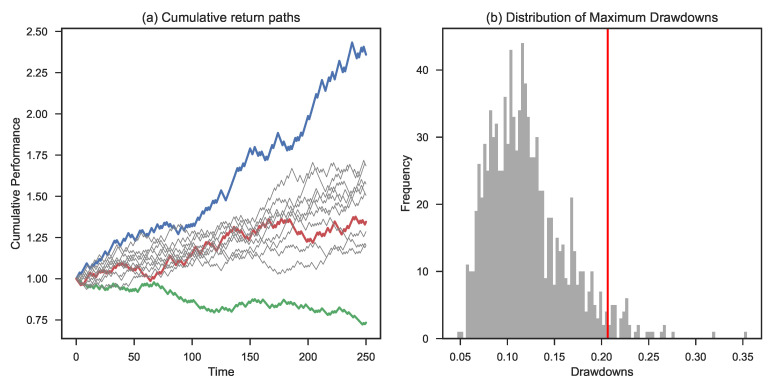
Simulation based on Bernoulli trials assuming a leverage factor of 1%. Panel (**a**) shows the highest (blue color), lowest (green color), and 50th percentile final wealth (red color) trajectory. Panel (**b**) shows the maximum drawdown distribution and the corresponding $CED_{0.1}$ (in red).

Figure 7 zooms in on the 1% initial risk level. Panel (a) shows the highest, lowest, and 50th percentile path (ranked based on final outcomes) along with a range of different simulated paths. Panel (b) provides the empirical distribution function of the maximum drawdown of each individual path. The red line shows the $CED_{0.1}$ level of 21%. This means that the average of the 10% worst maximum drawdowns equals 21%. The ensemble average of maximum drawdowns is 12% in this case.

#### 4.2.2. CASE II: Uniform Distribution

**Figure 8:**
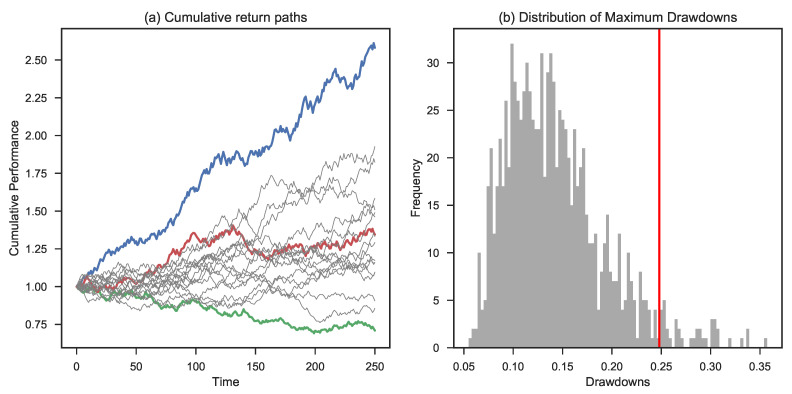
Simulation based on a uniform distribution for the return factor assuming an initial risk of 1%. Panel (**a**) shows the highest (in blue), lowest (in green), and 50th percentile final wealth trajectory (in red). Panel (**b**) shows the maximum drawdown distribution and the corresponding $CED_{0.1}$ (in red).

The results of the simulations for different levels of initial risk in the case of a uniform distribution can be found in Table 3 and Figure 8. At first sight, we note the median levels compared to the previous experiment drop slightly, and the maximum drawdown figures deteriorate.

Next, while keeping the expected return factor constant, we add extra randomness by assigning a small probability to higher absolute values in terms of both gains and losses:

(4) $$RF_{Win}∼\left\{\begin{array}{cc} U(0,2.5) & \mathrm{with}\;\mathrm{probability}\;0.47\\ U(2.5,6) & \mathrm{with}\;\mathrm{probability}\;0.03 \end{array}\right.$$

(5) $$RF_{Loss}∼\left\{\begin{array}{cc} U(-2,0) & \mathrm{with}\;\mathrm{probability}\;0.47\\ U(-6,-2) & \mathrm{with}\;\mathrm{probability}\;0.03 \end{array}\right.$$

**Figure A3:**
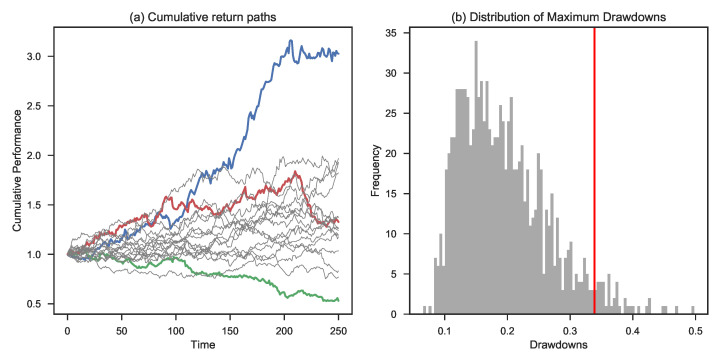
Simulation based on uniform distribution with outliers, assuming a leverage factor of 1%. Panel (**a**) shows the highest (in blue), lowest (in green), and 50th percentile final wealth trajectory (in red). Panel (**b**) shows the maximum drawdown distribution and the corresponding CED (in red).

The results, which can be found in Table A1 and Figure A3 in Appendix A.2, confirm the trend of lower overall return values and worse drawdown characteristics.

#### 4.2.3. CASE III: Exponential Distribution

**Figure 9:**
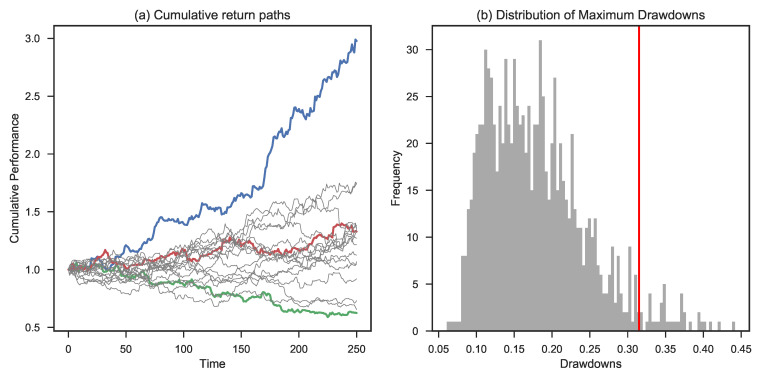
Simulation based on an exponential return factor with an initial risk of 1%. Panel (**a**) shows the highest (in green), lowest (in blue), and 50th percentile final wealth trajectory (in red). Panel (**b**) shows the maximum drawdown distribution and the corresponding $CED_{0.1}$ (in red).

Table 4 and Figure 9 show the results for the exponential distribution. In this case, we notice the impact of heavier tails. On the one hand, the optimal leverage is lower than in the first experiment linked to the uniform distribution. On the other hand, this results in a higher $CED_{0.1}$ measure.

#### 4.2.4. CASE IV: Lomax Distribution

**Figure 10:**
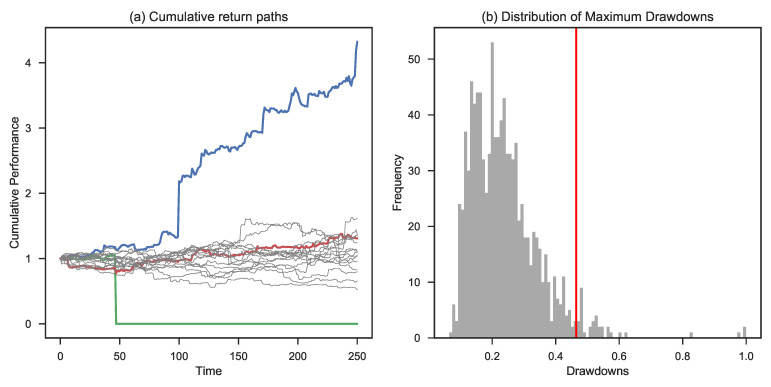
Simulation based on a Lomax distribution assuming a risk factor of 1%. Panel (**a**) shows the highest (in blue), lowest (in green), and 50th percentile final wealth trajectory (in red). Panel (**b**) shows the maximum drawdown distribution and the corresponding $CED_{0.1}$ (in red).

The results shown in Table 5 show that the moment the $IR_{i}$ exceeds 1%, the CED measure quickly moves to 75%. Figure 10 describes the evolution in the case of an $IR_{i}$ of 1%: already at this low level of leverage, we see the presence of ruin in the case of some extreme events.

### 4.3. Discussion

**Figure 11:**
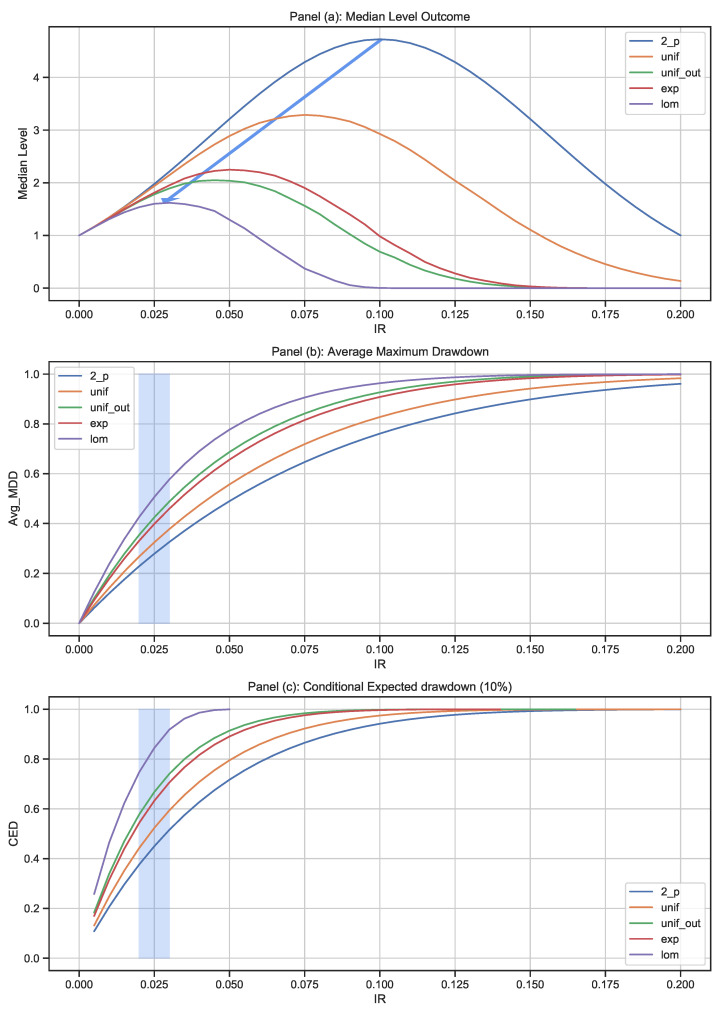
Comparison of the experiments: Panel (**a**) shows the median level outcome for the various simulations at different levels of IR; panels (**b**) and (**c**) show the associated average maximum drawdown levels and the conditional expected drawdown Level at a 10% confidence level.

Figure 11 provides a summary of the different experiments for the median level outcome, the average maximum drawdown, and the $CED_{0.1}$. This graph shows why cautiousness in the face of uncertainty is so important. First, we note that additional randomness and more extreme possible return factors lower the expectation for the median level outcome and lead to lower optimal IR taking. The blue arrow in panel (a) highlights the critical impact of tail risk within the outcome distribution. At 10% initial risk (IR), we obtain the highest median level for the 2-point distribution. For the uniform distribution with outliers, we note that the highest median level is achieved at an IR below 5%. The heaviest tailed distribution, represented by the Lomax distribution, reaches its peak growth at an IR level of around 2.5%. These observations are in line with the Kelly criterion and a direct consequence of the impact of randomness on the ability to assume risk and generate favorable outcomes.

Panels (b) and (c) in Figure 11 highlight the impact of different return distributions on drawdown risk. As an illustration, we zoom in on the IR area of 2.5% in panel (b). Assuming the risk-taker is conservative, we still observe a very big difference in terms of average maximum drawdown (AMDD). Our 2-point distribution has an AMDD of around 30%, whereas the more risky Lomax distribution has an AMDD of around 50%. These effects become even more pronounced when considering the tail of the drawdown distribution via the $CED_{0.1}$ measure. Panel (c) shows how these differences are further increased: the 2-point distribution has a $CED_{0.1}$ slightly above 40%, whereas for the Lomax distribution, the $CED_{0.1}$ is slightly above 80%.

A trading operation that takes an IR of 2.5%, assuming a uniform distribution with outliers for the return factor, would expect a resulting $CED_{0.1}$ of around 50%. However, in the case where the return factor follows a Lomax distribution, this would lead to a $CED_{0.1}$ measure of more than 80%. Assuming a distribution of outcomes and a maximum drawdown level will provide the investor with a target level of IR to take. Ideally, this would require a full understanding of the future return outcomes, which is not possible in practice. However, risk-takers can apply the reasoning behind these charts to their own investment process and consider what level of drawdown they are willing to bear.

The results obtained through this simulation exercise can be used to establish a maximum drawdown corridor along the lines of [36]. The upper boundary on the maximum drawdown can, for example, be defined as a level of maximum drawdown that can only be exceeded with a 5% chance. Equally, the lower level of the drawdown corridor can be defined as the maximum drawdown level that will not be reached with a probability of 5%.

## 5. Conclusions

Long losing streaks can have a dramatic psychological impact. An awareness that losing streaks of such length are to be expected could reduce the emotional impact when they happen and make sure the risk-taker adheres to a predetermined risk policy. While many papers related to bet sizing focus on final outcomes, we zoom in on the path that a risk-taker experiences. More specifically, we demonstrate the importance of drawdown risk in the case of repeated favorable opportunities. Rej et al. [36] point out that managers and investors tend to underestimate the length and depth of perfectly normal and acceptable drawdowns.

The occurrence of losing sequences and their magnitude plays a key role in the drawdown characteristics of the portfolio or account. The literature shows various misconceptions related to random sequences. Kahneman and Tversky [37] analyzed judgment under uncertainty and found through a series of empirical examples that people do not follow the principles of probability theory in judging the likelihood of uncertain events. A random unbiased coin-tossing process generates a similar amount of heads and tails over the long run. Intuition leads people to believe that this should equally be the case for short sequences (People, for example, judge sequences as more likely if there is some irregularity: for example, HTTTTHHT is deemed more likely than HTHTHTHT. Other examples of biases related to sequences are the “hot hand fallacy” and the “gambler’s fallacy”.).

Hahn and Warren [38] argue that people’s biases are reflective of their experience and that it should come as no surprise that people also seem to respond well to corrective experience in the form of training and feedback in generation and prediction tasks. These findings are consistent with the Copenhagen experiment, in which tested individuals exhibited greater risk aversion when shifting from additive to multiplicative dynamics [35,39].

Monte Carlo simulation allows for a comprehensive understanding of the impact of randomness by mathematically modeling potential outcomes. This allows for a visualization of how various paths can play out. In a thought experiment, we analyze performance paths for an operation with a positive edge based on various risk-taking considerations related to the risk taken per trade, the distribution of trade outcomes, and the probability of winning or losing.

This experiment highlights why being cautious in investing is so relevant. First, we show that the expected distribution function for return outcomes plays a major role in determining the initial risk to target to achieve the highest median level outcome. Secondly, we show the importance of tail risks for various drawdown metrics. A wrong assessment of tail risks can lead to a significant underestimation of drawdown risk.

How does one assess the parameters for these distribution functions? A historical record, which ideally went through different market regimes, can give a good first idea of the variables discussed above. Even without having a historical record, the analysis presented herein can be used to evaluate several possible risk-taking scenarios. Instead of looking solely at median level outcomes, risk-takers should consider these drawdown statistics and look at a combination of these metrics when determining ‘optimal’ risk in the case of an edge.

While we consider risk from a different perspective by studying drawdown behavior, our results generally confirm previous research: MacLean et al. [40] studied value at risk (VaR) constraints but also period-by-period drawdown constraints and found that at low levels of risk control, the capital growth or Kelly strategy is optimal. However, as risk control requirements tighten, the strategy should become more conservative. Lopez de Prado [28] shows that under a finite time horizon, the leverage suggested by the growth-optimal portfolio theory, needs to be adjusted down considerably. Also, [14] show how uncertainty about the true probability distribution of returns and the presence of left tail constraints are sufficiently powerful to override considerations in conventional theory. They therefore suggest using a “barbell portfolio” with one set of holdings with low risk and one set of holdings with very high risk.

Analyzing various outcomes through Monte Carlo simulation can assist in building a rational risk policy that can avoid psychological and financial headaches. The framework developed in this paper can lead to better-informed decisions for the risk-taker. An overly optimistic investor may dampen too high expectations by taking a look at a broad section of possible outcome paths.

The framework presented in this paper can enable risk-takers to understand better the possible maximum drawdown risks they face when following a certain investment strategy. This in turn can lead to the identification of possible risk of ruin in case position sizing and the resulting leverage is too large. A second advantage is that investors can compare the real-life performance of their investments or trading strategies versus the model output and can get an indication of whether their strategy is no longer performing. This could allow them to differentiate between an unlucky streak and possibly more worrisome events.

## Figures and Tables

**Table 1:** Distributions used in the different experiments.

Exp.	Distribution	$P_{w}$	${\mathrm{RF}}_{\mathrm{Win}}$	$P_{l}$	${\mathrm{RF}}_{\mathrm{Loss}}$
I	2-point	0.5	1.25	0.5	−1
II	Uniform	0.5	∼U(0,2.5)	0.5	∼U(0,−2)
III	Exponential	0.5	∼Exp(0.8)	0.5	∼−Exp(1)
IV	Lomax	0.5	∼Lomax(3,2.5)	0.5	∼−Lomax(3,2)

^1^ In a variation to this experiment, we add additional randomness by assigning 47% of the outcomes to *RF_Win_* ∼ *U*(0, 2.5) and 3% of the outcomes to *RF_Win_* ∼ *U*(2.5, 6); 47% of the outcomes to *RF_Loss_* ∼ *U*(0,−2) and 3% of the outcomes to *RF_Loss_* ∼ *U*(−2,−6) while keeping the expected return factor constant. ^2^ Lomax((α, λ): The Cumulative Distribution Function for the Lomax distribution is given by: $1-{\left[1+\frac{x}{\lambda }\right]}^{-\alpha }$, with shape parameter α and scale parameter λ.

**Table 2:** Final strategy statistics by leverage: two-point distribution.

	Strategy Returns				Strategy Maximum Drawdowns								
Lev	MinL	MedL	AvgL	MaxL	Min	Avg	Max	CED	>10%	>20%	>30%	>40%	>50%
0.5%	0.86	1.16	1.17	1.54	0.02	0.06	0.19	0.11	59	0	0	0	0
1.0%	0.73	1.35	1.36	2.36	0.05	0.12	0.35	0.21	661	46	2	0	0
2.0%	0.52	1.75	1.85	5.39	0.09	0.23	0.59	0.38	999	614	165	24	2
5.0%	0.16	3.21	4.60	52.69	0.22	0.49	0.91	0.72	1000	1000	966	751	428
10.0%	0.01	4.72	20.33	1250.63	0.43	0.76	0.99	0.94	1000	1000	1000	1000	989
15.0%	0.00	3.21	80.76	13,706.78	0.60	0.90	1.00	0.99	1000	1000	1000	1000	1000
25.0%	0.00	0.14	415.65	166,350.13	0.84	0.99	1.00	1.00	1000	1000	1000	1000	1000

**Table 3:** Final strategy statistics by leverage: uniform returns.

	Strategy Returns				Strategy Maximum Drawdowns								
Lev	MinL	MedL	AvgL	MaxL	Min	Avg	Max	CED	>10%	>20%	>30%	>40%	>50%
0.5%	0.85	1.17	1.17	1.62	0.03	0.07	0.20	0.1313	153	0	0	0	0
1.0%	0.71	1.34	1.38	2.58	0.05	0.14	0.36	0.2480	795	125	13	0	0
2.0%	0.48	1.73	1.89	6.36	0.11	0.27	0.60	0.4429	1000	762	305	77	19
5.0%	0.12	2.89	4.92	72.57	0.26	0.56	0.91	0.7951	1000	1000	991	880	645
10.0%	0.01	2.93	22.10	1740.69	0.47	0.83	1.00	0.9748	1000	1000	1000	1000	998
15.0%	0.00	1.11	73.78	14,108.52	0.67	0.94	1.00	0.9985	1000	1000	1000	1000	1000
25.0%	0.00	0.00	105.79	49,825.25	0.89	1.00	1.00	1.0000	1000	1000	1000	1000	1000

**Table 4:** Final strategy statistics by leverage: exponential return factor.

	Strategy Returns				Strategy Maximum Drawdowns								
Lev	MinL	MedL	AvgL	MaxL	Min	Avg	Max	CED	>10%	>20%	>30%	>40%	>50%
0.5%	0.80	1.16	1.17	1.74	0.03	0.09305	0.25	0.1696	359	15	0	0	0
1.0%	0.62	1.33	1.38	2.98	0.06	0.17899	0.44	0.3151	929	331	55	4	0
2.0%	0.37	1.66	1.90	8.34	0.12	0.33112	0.71	0.5441	1000	908	562	245	78
5.0%	0.05	2.25	4.94	128.54	0.29	0.65636	0.97	0.8908	1000	1000	999	976	834
10.0%	0.00	0.98	20.94	4088.56	0.54	0.90833	1.00	0.9966	1000	1000	1000	1000	1000
15.0%	0.00	0.03	58.98	33,011.19	0.78	0.98362	1.00	nan	1000	1000	1000	1000	1000
20.0%	0.00	0.00	60.58	49,048.94	0.91	0.99842	1.00	nan	1000	1000	1000	1000	1000

**Table 5:** Final strategy statistics by leverage: Lomax distribution.

	Strategy Returns				Strategy Maximum Drawdowns								
Lev	MinL	MedL	AvgL	MaxL	Min	Avg	Max	CED	>10%	>20%	>30%	>40%	>50%
0.5%	0.37	1.16	1.18	2.17	0.03	0.12	0.73	0.26	612	100	14	4	3
1.0%	0.00	1.31	1.38	4.32	0.06	0.24	1.00	0.46	974	583	222	74	22
2.0%	0.00	1.54	1.92	14.67	0.13	0.43	1.00	0.75	1000	966	750	525	282
5.0%	0.00	1.30	5.14	259.34	0.31	0.78	1.00	1.00	1000	1000	1000	993	933
10.0%	0.00	0.00	24.94	5463.56	0.61	0.96	1.00	nan	1000	1000	1000	1000	1000
15.0%	0.00	0.00	37.31	11,016.51	0.84	1.00	1.00	nan	1000	1000	1000	1000	1000
25.0%	0.00	0.00	0.01	12.59	0.99	1.00	1.00	nan	1000	1000	1000	1000	1000

**Figure A1:**
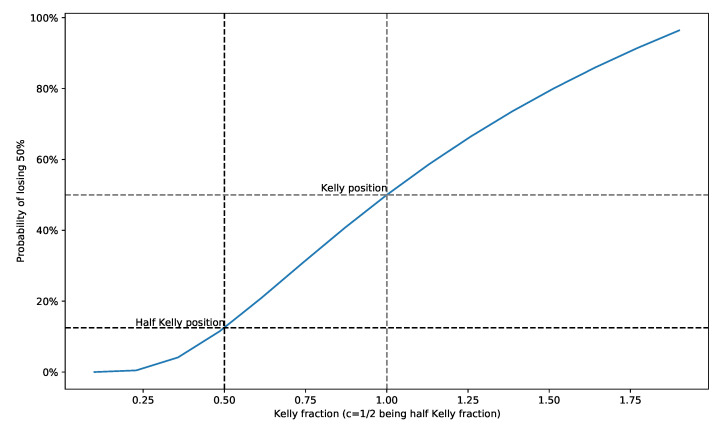
Impact of fractional Kelly on the probability of experiencing a 50% drawdown.

**Figure A2:**
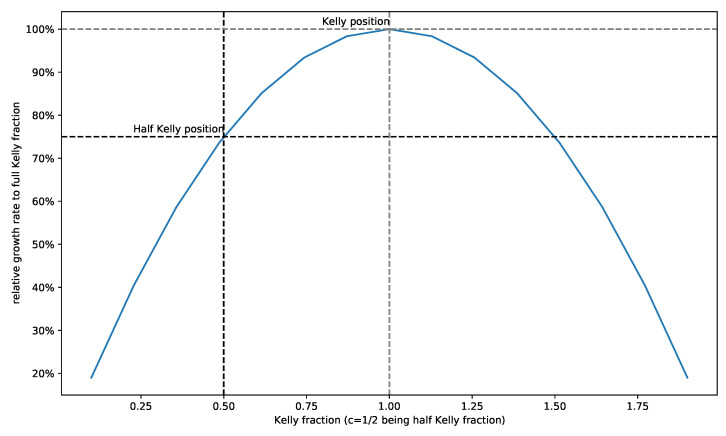
Impact of fractional Kelly on relative long term growth rates.

## Data Availability

Not applicable.

## Appendix A

### Appendix A.1. Drawdowns and the Kelly Criterion

Assuming a lognormal diffusion process, with drift *m*, variance $s^{2}$, and risk-free rate r=0:

(A1) $$\begin{array}{c} \begin{array}{c} f^{*}=m/s^{2},\\ E\left(G_{\infty }\left(f\right)\right)=g_{\infty }\left(f\right)=mf-s^{2}f^{2}/2,\\ g_{\infty }\left(f^{*}\right)=m^{2}/2s^{2},\\ Var(\left(G_{\infty }\left(f\right)\right)=s^{2}f^{2}, \end{array} \end{array}$$

where G measures the exponential rate of increase per trial, $f^{*}$ is the optimal fraction to invest, and $g_{\infty }$ is the expected asymptotic growth rate.

As the Kelly criterion and the optimal Kelly fraction $f^{*}$ lead to big drawdowns, the following formula shows the impact of fractional Kelly investing on the probability of experiencing a drop to fraction x of the initial capital. Setting $f=cf^{*}$ and V(t) the investment value after time t:

(A2) $$\begin{array}{c} \begin{array}{c} Prob\left(V\left(t\right)/V_{0}\leq x\;\mathrm{for}\;\mathrm{some}\;t\right)=x^{(2g_{\infty }/Var\left(G_{\infty }\right)} \end{array} \end{array}$$

Assuming for simplicity that $2g_{\infty }/Var\left(G_{\infty }\right)=1$, the probability of halving in value (x=0.5) equals $x^{1}$, so 50%.

To determine the impact of experiencing a big drawdown for a partial Kelly position, one can set $f=cf^{*}$, where *c* is a fraction of the optimal $f^{*}$. For comparative risk and again under the assumption that $2g_{\infty }/Var\left(G_{\infty }\right)=1$, the following formula can be used:

(A3) $$\begin{array}{c} \begin{array}{c} Prob\left(V\left(t\right)/V_{0}\leq x\;\mathrm{for}\;\mathrm{some}\;t\right)=x^{\left(2/c-1\right)} \end{array} \end{array}$$

Figure A1 shows the impact of reducing the Kelly fraction on the probability of having a loss greater than 50%.

The relationship between fractional growth ($g_{\infty }\left(cf^{*}\right)$) and Kelly growth ($g_{\infty }\left(f^{*}\right)$) is given by:

(A4) $$\begin{array}{c} g_{\infty }\left(cf^{*}\right)/g_{\infty }\left(f^{*}\right)=c\left(2-c\right) \end{array}$$

This relationship is illustrated in Figure A2. It shows that by assuming a half Kelly position, the investor’s long-term growth rate drops by 25%.

### Appendix A.2. Uniform Distribution with Outliers

**Table A1:** Final strategy statistics by leverage: uniform returns with outliers.

	Strategy Returns				Strategy Maximum Drawdowns								
**Lev**	**MinL**	**MedL**	**AvgL**	**MaxL**	**Min**	**Avg**	**Max**	**CED**	**>10%**	**>20%**	**>30%**	**>40%**	**>50%**
0.5%	0.74	1.16	1.17	1.76	0.03	0.10	0.29	0.18	431	20	0	0	0
1.0%	0.53	1.33	1.37	3.03	0.07	0.19	0.50	0.34	969	403	84	9	0
2.0%	0.26	1.65	1.89	8.50	0.13	0.35	0.77	0.58	1000	962	628	315	110
5.0%	0.02	2.04	5.08	122.00	0.32	0.69	0.99	0.91	1000	1000	1000	991	896
10.0%	0.00	0.69	23.34	2346.58	0.58	0.93	1.00	1.00	1000	1000	1000	1000	1000
15.0%	0.00	0.02	35.94	4763.24	0.78	0.99	1.00	1.00	1000	1000	1000	1000	1000
25.0%	0.00	0.00	1.51	686.72	0.98	1.00	1.00	nan	1000	1000	1000	1000	1000

## References

[1] Markowitz H. (1952). Portfolio Selection. J. Financ..

[2] Kelly J.L. (1956). A new interpretation of the information rate. Bell Syst. Tech. J..

[3] Latane H.A. (1959). Criteria for choice among risky ventures. J. Political Econ..

[4] Markowitz H.M. (1959). Portfolio Selection, Cowles Foundation Monograph 16.

[5] Thorp E.O. (1969). Optimal gambling systems for favorable games. Rev. L’Institut Int. Stat..

[6] Thorp E.O. (2011). Understanding the Kelly criterion. The Kelly Capital Growth Investment Criterion: Theory and Practice.

[7] MacLean L.C., Ziemba W.T., Blazenko G. (1992). Growth versus security in dynamic investment analysis. Manag. Sci..

[8] Maclean L., Thorp E., Ziemba W. (2010). Long-term capital growth: The good and bad properties of the Kelly and fractional Kelly capital growth criteria. Quant. Financ..

[9] Peters O. (2011). Optimal leverage from non-ergodicity. Quant. Financ..

[10] Peters O., Gell-Mann M. (2016). Evaluating gambles using dynamics. Chaos Interdiscip. J. Nonlinear Sci..

[11] Thorp E.O. (1971). Portfolio Choice and the Kelly Criterion. Proc. Bus. Econ. Sect. Am. Stat. Assoc..

[12] Bernoulli D. (1954). Exposition of a new theory on the measurement. Econometrica.

[13] Spitznagel M. (2021). Safe Haven: Investing for Financial Storms.

[14] Geman D., Geman H., Taleb N.N. (2015). Tail risk constraints and maximum entropy. Entropy.

[15] Allen D.E., McAleer M., Powell R.J., Singh A.K. (2016). Down-Side Risk Metrics as Portfolio Diversification Strategies across the Global Financial Crisis. J. Risk Financ. Manag..

[16] MacLean L.C., Thorp E.O., Zhao Y., Ziemba W.T. (2011). Medium Term Simulations of The Full Kelly and Fractional Kelly Investment Strategies. The Kelly Capital Growth Investment Criterion, World Scientific Handbook in Financial Economics Series.

[17] Geboers H., Depaire B., Annaert J. (2022). A review on drawdown risk measures and their implications for risk management. J. Econ. Surv..

[18] Möller P.M. (2018). Drawdown Measures and Return Moments. Int. J. Theor. Appl. Financ..

[19] Chekhlov A., Uryasev S., Zabarankin M. (2005). Drawdown measure in portfolio optimization. Int. J. Theor. Appl. Financ..

[20] Zabarankin M., Pavlikov K., Uryasev S. (2014). Capital Asset Pricing Model (CAPM) with drawdown measure. Eur. J. Oper. Res..

[21] Ding R., Uryasev S. (2022). Drawdown beta and portfolio optimization. Quant. Financ..

[22] Goldberg L.R., Mahmoud O. (2017). Drawdown: From practice to theory and back again. Math. Financ. Econ..

[23] Thorp E.O. (2011). The Kelly Criterion in Blackjack Sports Betting, and the Stock Market. The Kelly Capital Growth Investment Criterion, World Scientific Handbook in Financial Economics Series.

[24] Wu M.E., Lin S.H., Wang J.C. (2020). Embedded draw-down constraint using ensemble learning for stock trading. J. Intell. Fuzzy Syst..

[25] Maier-Paape S. (2018). Risk averse fractional trading using the current drawdown. J. Risk.

[26] Maslov S., Zhang Y.C. (1999). Probability distribution of drawdowns in risky investments. Phys. A Stat. Mech. Its Appl..

[27] Poundstone W. (2010). Fortune’s Formula: The Untold Story of the Scientific Betting System That Beat the Casinos and Wall Street.

[28] Lopez de Prado M. (2013). How Long Does It Take to Recover from a Drawdown? SSRN Scholarly Paper ID 2254668.

[29] Mercurio P.J., Wu Y., Xie H. (2020). Option portfolio selection with generalized entropic portfolio optimization. Entropy.

[30] Tran S., Verhoeven P. (2021). Kelly Criterion for Optimal Credit Allocation. J. Risk Financ. Manag..

[31] Harré M.S. (2022). Entropy, Economics, and Criticality. Entropy.

[32] Baz J., Guo H. (2017). An Asset Allocation Primer: Connecting Markowitz, Kelly, and Risk Parity. PIMCO.

[33] Carta A., Conversano C. (2020). Practical Implementation of the Kelly Criterion: Optimal Growth Rate, Number of Trades, and Rebalancing Frequency for Equity Portfolios. Front. Appl. Math. Stat..

[34] Lleo S., Ziemba W.T. (2015). The Swiss black swan bad scenario: Is Switzerland another casualty of the Eurozone crisis?. Int. J. Financ. Stud..

[35] Peters O. (2019). The ergodicity problem in economics. Nat. Phys..

[36] Rej A., Seager P., Bouchaud J.-P. (2018). You are in a drawdown. When should you start worrying?. Wilmott.

[37] Kahneman D., Tversky A. (1972). Subjective probability: A judgment of representativeness. Cogn. Psychol..

[38] Hahn U., Warren P.A. (2009). Perceptions of randomness: Why three heads are better than four. Psychol. Rev..

[39] Meder D., Rabe F., Morville T., Madsen K.H., Koudahl M.T., Dolan R.J., Siebner H.R., Hulme O.J. (2019). Ergodicity-breaking reveals time optimal decision making in humans. arXiv.

[40] MacLean L.C., Sanegre R., Zhao Y., Ziemba W.T. (2004). Capital Growth with Security. J. Econ. Dyn. Control.

